# Supplementary material for: Tumor Tissue-Specific Biomarkers of Colorectal Cancer by Anatomic Location and Stage
Source: Metabolites. 2020 Jun 19;10(6):257. doi: 10.3390/metabo10060257 (PMC7345993; doi:10.3390/metabo10060257)
Supplement: Supplementary file 1 [file metabolites-10-00257-s001.pdf]

Supplementary Information for

Tumor Tissue-Specific Biomarkers of Colorectal Cancer by Anatomic Location and Stage

Authors

Yuping Cai, Nicholas J.W. Rattray, Qian Zhang, Varvara Mironova, Alvaro Santos-Neto, Engjel Muca Ana K. Rosen Vollmar, Kuo-Shun Hsu, Zahra Rattray, Justin R. Cross, Yawei Zhang, Philip B. Paty, Sajid A. Khan, Caroline H. Johnson

Sajid A. Khan, Department of Surgery, Division of Surgical Oncology, Yale University School of Medicine, New Haven, CT, USA.

sajid.khan@yale.edu

Caroline H. Johnson, Department of Environmental Health Sciences, Yale School of Public Health, Yale University, New Haven, CT USA

caroline.johnson@yale.edu

Table S1. Demographics of colon cancer patients from samples used for discovery of metabolic differences between colon cancer and normal controls.

	**Normal Controls (n = 39)**	**Colon Cancer Discovery (n = 39)**	**Colon Cancer Validation (n = 39)**
**Sex, n**			
Male	27 (69%)	20 (51%)	20 (51%)
Female	12 (31%)	19 (49%)	19 (49%)
**Age, mean (sd)**	67.4 (12.4)	69.5 (8.5)	68.9 (6.8)
**Stage (I/II/III)**	——	13/14/12	13/14/12

Table S2. **List of 28 identified tumor tissue-specific metabolite biomarkers of colon cancer.**

**Metabolite**	**m/z (Da)**	**RT (seconds)**	**VIP**	**FC[a]**	**q value[b]**	**LC**	**Polarity**	**MSI[c]**
Taurine	124.0061	284.7	3.16	3.22	1.46E-14	HILIC	NEG	1
Glutamate	146.0446	385.7	2.43	2.83	9.02E-08	HILIC	NEG	1
CDP-choline	487.1012	431.5	2.22	6.18	5.65E-10	HILIC	NEG	1
Fructose 6-phosphate	241.0123	426.2	2.16	6.07	1.02E-09	HILIC	NEG	1
Hypoxanthine	119.0356	163.4	2.12	1.40	2.79E-07	HILIC	POS	1
Phenylalanine	207.1130	252.0	2.10	2.52	2.95E-06	HILIC	POS	2
Phosphoethanolamine	164.0079	441.2	2.06	2.23	5.85E-06	HILIC	POS	1
Creatinine	114.0655	164.3	2.04	0.34	7.44E-08	HILIC	POS	1
GDP-glucose	621.0890	434.0	2.01	4.22	2.47E-04	HILIC	NEG	1
CDP-ethanolamine	445.0537	436.0	2.00	6.37	5.76E-07	HILIC	NEG	1
Lactate	89.02333	218.2	1.94	1.58	5.26E-05	HILIC	NEG	1
Uracil	111.0204	88.3	1.93	2.60	2.07E-07	HILIC	NEG	1
Palmitoylcarnitine	400.3441	164.9	1.86	1.96	2.60E-05	HILIC	POS	2
S-adenosyl-L-homocysteine	385.1282	363.2	1.84	3.42	5.01E-06	HILIC	POS	1
PE (34:1)	716.5262	635.2	1.83	2.84	2.15E-05	RPLC	NEG	2
Fucose-1-phosphate	243.0285	438.0	1.80	2.65	9.68E-07	HILIC	NEG	1
CMP-N-acetylneuraminate	613.1417	434.1	1.80	2.48	1.45E-05	HILIC	NEG	1
UDP-N-acetylglucosamine	606.0746	423.6	1.78	8.93	1.28E-03	HILIC	NEG	1
GMP	362.0514	432.3	1.78	2.54	4.58E-05	HILIC	NEG	1
AMP	346.0567	399.4	1.78	1.70	1.02E-04	HILIC	NEG	1
dAMP	332.0761	402.3	1.69	3.34	1.10E-09	HILIC	POS	1
Glucose 6-phosphate	259.0227	464.0	1.65	3.60	1.10E-08	HILIC	NEG	1
LysoPE (22:5)	548.2712	425.3	1.61	0.35	1.63E-03	RPLC	NEG	2
UDP	384.9866	423.4	1.59	2.63	2.78E-04	HILIC	NEG	1
Formylmethionine	176.0393	188.9	1.58	2.05	4.45E-08	HILIC	NEG	1
Glycerol 3-phosphate	155.0099	433.9	1.55	0.58	2.68E-04	RPLC	POS	2
UDP -glucuronate	579.0289	469.1	1.54	3.78	6.75E-04	HILIC	NEG	1
Adenine	136.0610	394.4	1.50	1.53	3.40E-03	HILIC	POS	1

[a]**FC**, fold change was calculated by dividing median value of cancer samples by median value of normal samples; [b]**q** value was calculated using pairwise Wilcoxon Mann-Whitney U test with Benjamini-Hochberg-based FDR correction; [c]**MSI** metabolite standard initiative level [1].

Table S3. Demographics of colon cancer patients from samples used for discovery of metabolic differences between RCC and LCC. Right-sided colon cancer = RCC, Left-sided colon cancer = LCC.

		Stage I			Stage II			Stage III		
	Normal	RCC (n=22)	LCC (n=25)	P [a]	RCC (n=44)	LCC (n=42)	P [a]	RCC (n=32)	LCC (n=32)	P [a]
**Sex, n**										
Male	27(69%)	10(45%)	15 (40%)		23(52%)	25(59%)		15(47%)	14(44%)	
Female	12(31%)	12(55%)	10(60%)		21(48%)	17(41%)		17(53%)	18(56%)	
**Tumor size** mean (sd)		3.9 (1.5)	3.5 (1.3)	0.28	6.2 (2.6)	4.7 (1.7)	0.002	5.4 (1.8)	4.6 (1.7)	0.09
**Age, mean**										
Male	69.3	73.9	69.3		72.9	72.2		73.5	63.7	
Female	63.3	72.1	69.6		73.5	69.1		72.2	71.1	

[a] P value: Student *t* test

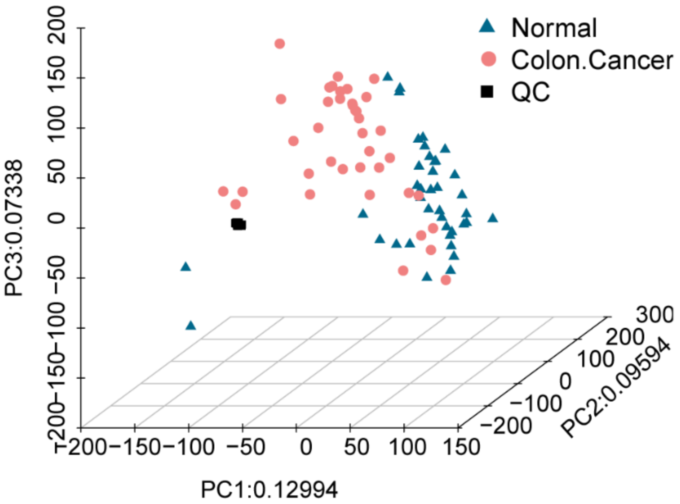

Figure S1: PCA scores plot from metabolomics analysis of normal colon tissues, colon cancer tissues, and quality control (QC) samples.

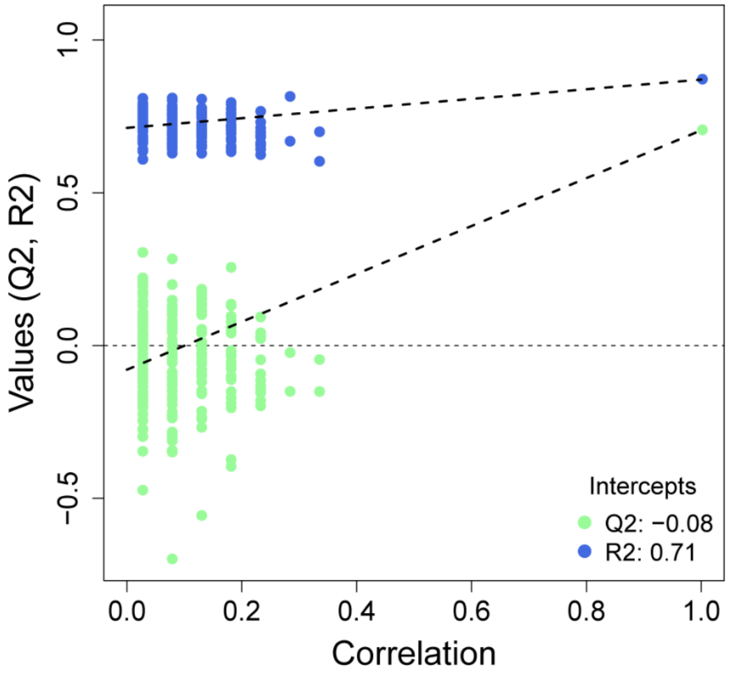

Figure S2: OPLS-DA validation plot obtained from 200 permutation tests on normal colon (n=39) and colon cancer samples (n=39).

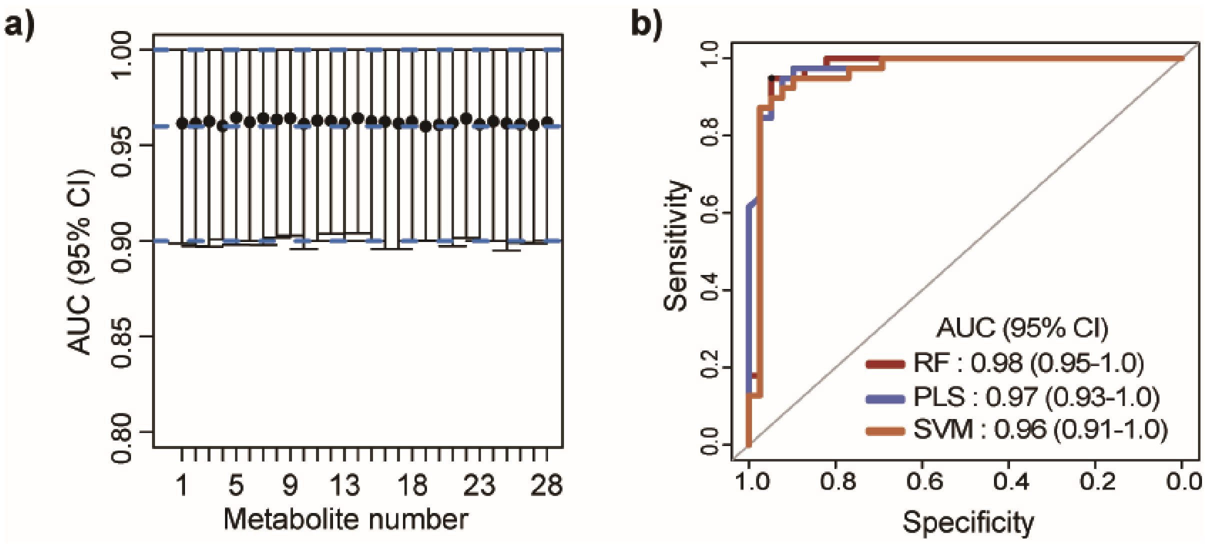

Figure S3: **(a)** AUC values of PLS prediction models with increasing number of metabolic biomarkers. **(b)** ROC plots of predictive models based on nine metabolic biomarkers using random forest (RF), partial least-squares (PLS), and support vector machine (SVM), respectively.

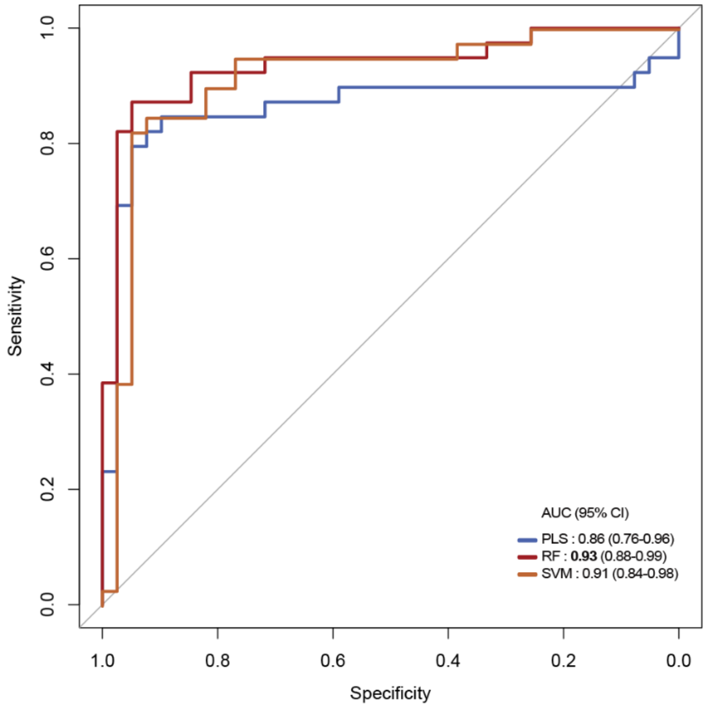

Figure S4: ROC plots of models in validation data set based on nine metabolic biomarkers using random forest (RF), partial least-squares (PLS), and support vector machine (SVM), respectively.

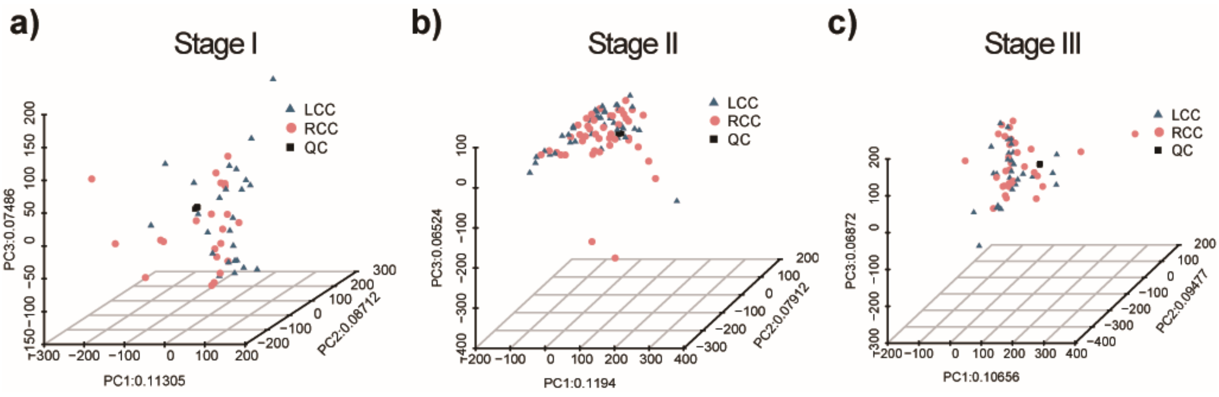

Figure S5: Stage stratified PCA score plots from left-sided colon cancer (LCC), right-sided colon cancer (RCC) tissues, and quality control (QC) samples.

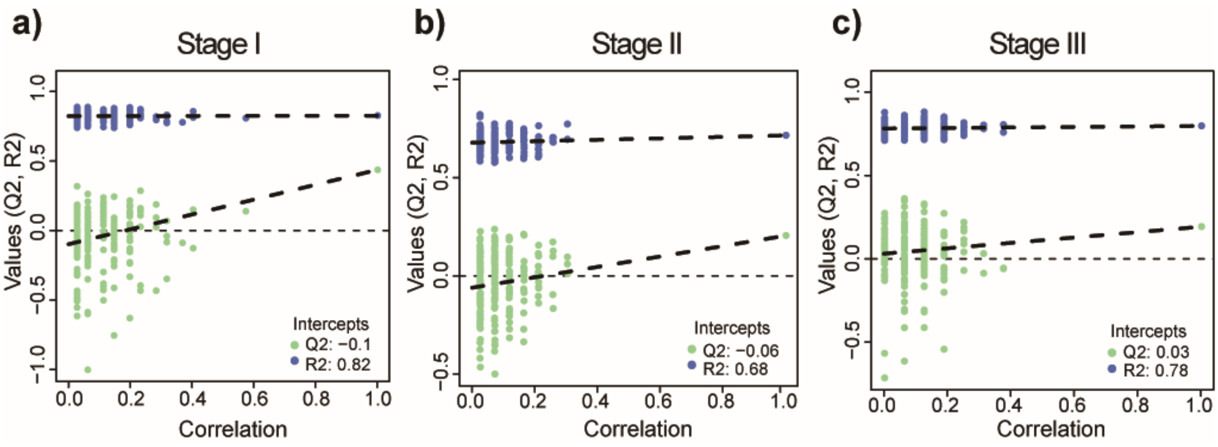

Figure S6: Validation plots obtained from 200 permutation tests for **(a)** stage I LCC (n = 25) and stage I RCC (n = 22) **(b)** stage II LCC (n = 42) and stage II RCC (n = 44), and **(c)** stage III LCC (n = 32) and stage III RCC (n = 32).

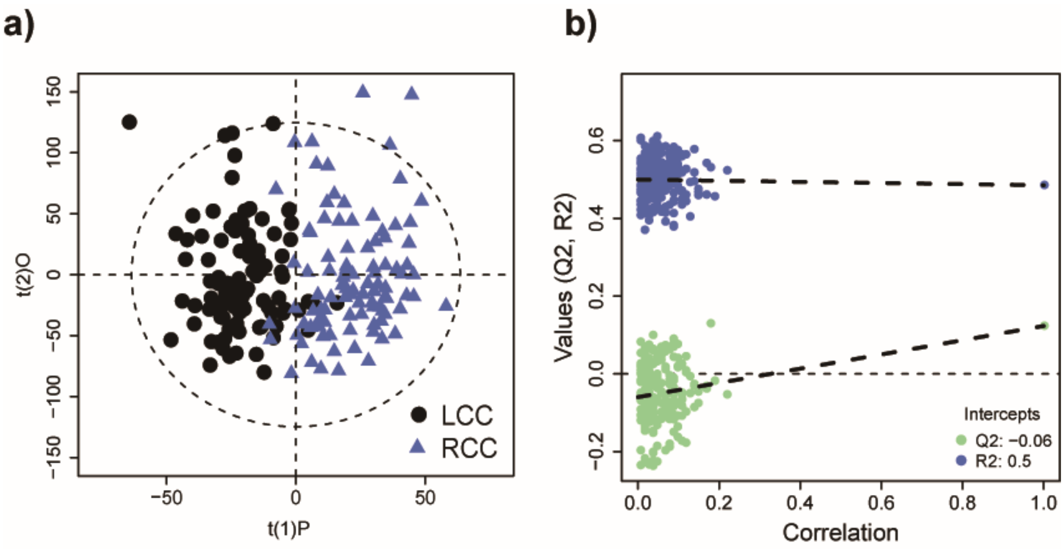

Figure S7: **(a)** OPLS-DA plot for metabolic signatures of stages I-III LCC (n = 99) and stages I-III RCC (n = 98). **(b)** Validation plots obtained from 200 permutation tests.

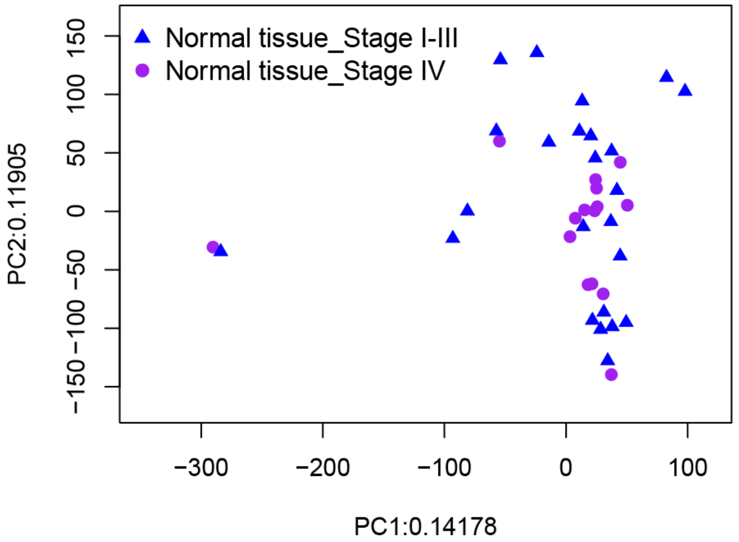

Figure S8: PCA scores plot of normal colon tissues from stage I-III patients and stage IV patients.

Reference:

1. Sumner, L.W.; Amberg, A.; Barrett, D.; Beale, M.H.; Beger, R.; Daykin, C.A.; Fan, T.W.; Fiehn, O.; Goodacre, R.; Griffin, J.L., *et al.* Proposed minimum reporting standards for chemical analysis chemical analysis working group (cawg) metabolomics standards initiative (msi). *Metabolomics : Official journal of the Metabolomic Society* **2007**, *3*, 211-221.
